# IL-17A injury to retinal ganglion cells is mediated by retinal Müller cells in diabetic retinopathy

**DOI:** 10.1038/s41419-021-04350-y

**Published:** 2021-11-08

**Authors:** Ao-Wang Qiu, Da-Rui Huang, Bin Li, Yuan Fang, Wei-Wei Zhang, Qing-Huai Liu

**Affiliations:** 1 Department of Ophthalmology, The First Affiliated Hospital of Nanjing Medical University, 300 Guangzhou Road, 210029 Nanjing, China; 2 Women & Children Central Laboratory, The First Affiliated Hospital of Nanjing Medical University, 300 Guangzhou Road, 210029 Nanjing, China

**Keywords:** Cell death, Medical research

## Abstract

Diabetic retinopathy (DR), the most common and serious ocular complication, recently has been perceived as a neurovascular inflammatory disease. However, role of adaptive immune inflammation driven by T lymphocytes in DR is not yet well elucidated. Therefore, this study aimed to clarify the role of interleukin (IL)-17A, a proinflammatory cytokine mainly produced by T lymphocytes, in retinal pathophysiology particularly in retinal neuronal death during DR process. Ins2^Akita^ (Akita) diabetic mice 12 weeks after the onset of diabetes were used as a DR model. IL-17A-deficient diabetic mice were obtained by hybridization of IL-17A-knockout (IL-17A-KO) mouse with Akita mouse. Primarily cultured retinal Müller cells (RMCs) and retinal ganglion cells (RGCs) were treated with IL-17A in high-glucose (HG) condition. A transwell coculture of RGCs and RMCs whose IL-17 receptor A (IL-17RA) gene had been silenced with IL-17RA-shRNA was exposed to IL-17A in HG condition and the cocultured RGCs were assessed on their survival. Diabetic mice manifested increased retinal microvascular lesions, RMC activation and dysfunction, as well as RGC apoptosis. IL-17A-KO diabetic mice showed reduced retinal microvascular impairments, RMC abnormalities, and RGC apoptosis compared with diabetic mice. RMCs expressed IL-17RA. IL-17A exacerbated HG-induced RMC activation and dysfunction in vitro and silencing IL-17RA gene in RMCs abolished the IL-17A deleterious effects. In contrast, RGCs did not express IL-17RA and IL-17A did not further alter HG-induced RGC death. Notably, IL-17A aggravated HG-induced RGC death in the presence of intact RMCs but not in the presence of RMCs in which IL-17RA gene had been knocked down. These findings establish that IL-17A is actively involved in DR pathophysiology and particularly by RMC mediation it promotes RGC death. Collectively, we propose that antagonizing IL-17RA on RMCs may prevent retinal neuronal death and thereby slow down DR progression.

## Introduction

Diabetic retinopathy (DR) is the most common and serious ocular complication [1]. In spite of the extensive research, the complex pathogenesis of DR has not been fully elucidated. For many years, it has been thought that DR manifests only with microangiopathic lesions, which are totally responsible for the loss of vision in diabetic patients [2]. However, in view of the current knowledge that during diabetes complex microvascular, neuronal, and glial abnormalities progressively disrupt retinal function and develop DR, DR is perceived as a neurovascular inflammatory disease [3–6]. The presence of high levels of inflammatory cytokines in the eye fluids of patients with DR has been observed, demonstrating a close correlation between inflammation and DR [7]. Nevertheless, the present knowledge is basically limited to the role of inflammation driven by retinal glial cells (Müller cells, astrocytes and microglia) in DR. The role of adaptive immune reaction driven by T lymphocytes in DR and its underlying mechanism remain poorly understood.

IL-17A is a proinflammatory cytokine produced mainly by T-helper 17 (Th17) cells, a subset of T lymphocytes. Initial studies indicate increased secretion of IL-17A from activated peripheral blood T cells in children with type 1 diabetes (T1D) [8] and a disorder in IL-17A that may have a significant impact on the course of autoimmune diabetes [9]. We previously showed that IL-17A injection in the vitreous cavity exacerbated diabetes-induced retinal microvascular lesions, activation and dysfunction of retinal Müller cells (RMCs), and apoptosis of retinal ganglion cells (RGCs) and that blocking IL-17A alleviated the diabetes-induced retinal abnormalities [10, 11]. Recently, Sigurdardottir et al. have found IL-17A-expressing T cells in the retinal vasculature and diabetes-mediated retinal inflammation, oxidative stress, and vascular leakage are all significantly lower in IL-17A^−/−^ mice [12]. These other and our findings suggest an involvement of IL-17A in DR pathophysiology. Further evidence needs to be provided to elucidate the mechanism by which IL-17A affects DR.

IL-17A signals through a heterodimeric receptor complex that consists of IL-17 receptor A (IL-17RA) and IL-17RC to affect cell function and survival [13]. Rat Müller cell line (rMC-1) expresses IL-17RA and anti-IL-17RA-neutralizing antibody reduces high-glucose (HG)-induced rMC-1 dysfunction [11]. In addition, IL-17RA-linking Act1-TRAF6-IKK-NFκB signaling pathway is involved in IL-17A injury to RMCs in DR [10]. By comparison, the expression of IL-17 receptors in neurons is controversial [14–17]. Retinal neurons include RGCs, amacrine cells, horizontal cells, bipolar cells, and light-sensitive photoreceptors [5]. In the retinal neurons, RGCs represent the best-studied neurons with respect to the effects of diabetes [18]. However, whether RGCs express IL-17 receptors and how IL-17A affects RGC survival are not well known. We hypothesized that RMCs may mediate IL-17A damage to RGCs in DR progression. RMCs are principal glia of the retina. They are the only cells to span the entire width of the retina, extending from ganglion cell layer (GCL) to photoreceptor inner segment area, and have intimate contact with both retinal blood vessels and retinal neurons [19, 20]. RMCs participate in formation of the blood–retinal barrier, regulate retinal glutamate metabolism, and support retinal neuronal survival [21, 22]. RMC activation, as demonstrated by an upregulation of glial fibrillary acidic protein (GFAP), has been identified in retinas of both diabetic patients and diabetic rodents in early stages of DR [23–25]. RMC-derived vascular endothelial growth factor (VEGF) accumulation results in diabetic retinal neovascularization and vascular leakage [26]. Hyperglycemia-induced decreases in both glutamine synthetase (GS) and excitatory amino acid transporter 1 (EAAT1) in RMCs may trigger apoptosis of RGCs in DR because of excitotoxicity caused by excessive glutamate [27, 28]. The structural and functional characteristics of RMCs constitute a foundation for transmitting IL-17A inflammatory information in DR development.

Accordingly, in this study, Ins2^Akita^ (Akita) mice that carry a spontaneous mutation in insulin 2 (Ins2) gene and develop insulin-dependent T1D were used as a DR model (Ins2^+/−^ genotype). IL-17A-knockout (IL-17A-KO) Akita mice (Ins2^+/−^/IL-17A^−/−^ genotype), which were obtained by crossbreeding of IL-17A-KO mouse with Ins2-mutant mouse, were employed to assess the effect of global IL-17A gene ablation on DR pathophysiology. Next, primarily cultured RMCs and RGCs were treated with IL-17A, respectively, in HG condition to show its effects on the cell function and survival. Finally, a transwell coculture of RGCs and IL-17RA-silenced RMCs was exposed to IL-17A in HG condition to determine whether RMCs mediate IL-17A injury to RGCs. The present study provides further evidence for IL-17A participation in DR pathogenesis and reveals a mechanism by which IL-17A promotes DR progression.

## Materials and methods

### IL-17A-deficient Akita mice

Akita mice on a C57BL/6 background develop insulin-dependent diabetes with the characteristic of robust hyperglycemia at the age of 4 weeks [29, 30] and exhibit evident DR pathology 10–14 weeks after hyperglycemia [31]. Akita mice of Ins2^+/−^ genotype were purchased from Model Animal Research Center of Nanjing University, Nanjing, China. Male Ins2^+/−^ mice 12 weeks after diabetic onset were used as DR model. Age-matched male wild-type (WT) littermates on a C57BL/6 genetic background served as a control of DR model. IL-17A-KO (IL-17A^−/−^ genotype) mice on a C57BL/6 background were kindly provided by Dr. Yang S. (Nanjing Medical University, Nanjing, China). IL-17A-deficient Akita mice were prepared by crossbreeding of Ins2^+/−^ genotype mice with IL-17A^−/−^ genotype mice as described in our previous publication [32]. First, female Ins2^+/−^ mice were crossed with male IL-17A^−/−^ mice and heterozygous mice of Ins2^+/−^/IL-17A^+/−^ genotype were obtained in F1 generation. Next, female Ins2^+/−^/IL-17A^+/−^ mice were crossed with male IL-17A^−/−^ mice and Ins2^+/−^/IL-17A^−/−^ genotype mice were obtained in F2 generation. Male Ins2^+/−^/IL-17A^−/−^ mice at the age of 16 weeks were collected as IL-17A-deficient DR model. Age-matched male Ins2^+/−^ siblings were used as a control of Ins2^+/−^/IL-17A^−/−^ mice. The WT mice, Akita mice, and IL-17A-deficient Akita mice were randomly allocated to the different experiments. The genotypes of mice were identified at 3–4 weeks of age by polymerase chain reaction (PCR) amplification.

All animal procedures were conducted in compliance with the Association for Research in Vision and Ophthalmology Statement for the Use of Animals in Ophthalmic and Vision Research and were approved by the Institutional Animal Care and Use Committees of Nanjing Medical University.

### Retinal vascular leukostasis

Retinal leukostasis was performed as described previously [10]. Mice were anesthetized by isoflurane inhalation and perfused through the left heart ventricle with 10 ml phosphate-buffered saline (PBS) to remove erythrocytes and non-adherent leukocytes. The mice were perfused with fluorescein isothiocyanate (FITC)-conjugated concanavalin A (Vector Laboratories, Burlingame, CA, USA) to label adherent leukocytes, followed by 10 ml PBS perfusion to flush out unbound concanavalin A. The eyes were fixed in 4% paraformaldehyde for 1 h and the retinas were flat mounted. Luminal leukocytes were counted by an investigator blinded to different groups. Total number of adherent leukocytes in vessels per retina was statistically analyzed.

### Retinal angiography

Retinal angiography was conducted as described previously [10]. Mice were anesthetized and perfused through the left ventricle with 1 ml PBS that contained FITC-dextran (Sigma-Aldrich, St. Louis, MO, USA). The eyes were enucleated and fixed in 4% paraformaldehyde for 1 h. The retinas were dissected, flat mounted, and viewed by fluorescence microscopy.

### Primary RMC culture and treatments

C57BL/6 mice at postnatal days 6–7 were sacrificed by cervical dislocation and the eyes were enucleated into D-hank’s buffer (Gibco, Rockville, MD, USA). The dissected retinas were incubated for 30 min in D-hank’s buffer supplemented with collagenase and trypsin, followed by addition of Dulbecco’s Modified Eagle Medium (DMEM) containing 10% fetal bovine serum (Gibco, Rockville, MD, USA) to terminate the digestion. The tissue microaggregates and single cells were seeded in culture flasks at a density of 0.7–1 retina/cm^2^ and maintained in DMEM containing 5 mM glucose (as a control). After 3 days, the retinal aggregates and debris were removed by vigorous rinsing, and the cells were kept in the culture for 7–10 days, leaving a purified cell monolayer of RMCs. The cells were incubated in DMEM containing 25 mM glucose (as a HG treatment) for 48 h to mimic diabetes in vitro. Recombinant IL-17A (50 ng/ml; R&D Systems, Minneapolis, MN, USA) was applied to HG-treated RMCs for 24 h. An adenoviral vector that expressed short hairpin RNA targeting IL-17RA (IL-17RA-shRNA) was constructed, amplified, and purified by Hanbio Biotechnology in Shanghai, China. IL-17RA-shRNA at a multiplicity of infection of 20 infected primary RMCs in HG medium, and Scr-shRNA was used as a control. After infection for 24 h, the cells were cultured in fresh medium for an additional 24 h before harvest.

### Primary RGC culture and treatments

Primary RGCs were purified by sequential immunopanning as described previously [33, 34] with minor modifications. Dissected retinas from 5- to 7-day-old C57BL/6 mice were digested with papain (160 U/ml; Worthington, Lakewood, NJ, USA) and DNase (200 U/ml; Sigma-Aldrich, St. Louis, MO, USA) at 37 °C for 30 min, which was terminated by rinsing the cells in buffers containing trypsin inhibitor. The retinal cells were resuspended in panning buffer, filtered through a 20 μm nylon mesh, and incubated with rat anti-macrophage antibody (Abcam, Cambridge, UK). Contaminating microglial cells were removed by incubating the cells on an anti-rat IgG-coated dish, followed by RGC selection on dishes sequentially coated with goat anti-mouse IgG and mouse anti-Thy1.2 (clone F7D5; AbD Serotec, Oxford, UK). The RGCs were kept in the culture of Neurobasal medium supplemented with glucose (5 mM), penicillin (100 unit/ml), streptomycin (100 μg/ml), insulin (0.5 μg/ml), sodium pyruvate (1.5 mM), 3,3′,5-Triiodo-l-thyronine (40 ng/ml), L-glutamine (2 mM), B27 (2%), N2 (2%), brain-derived neurotrophic factor (50 ng/ml), ciliary neurotrophic factor (10 ng/ml), and forskolin (10 μM) for 5 days. The culture medium and supplemented reagents were purchased from Sigma-Aldrich, St. Louis, MO, USA; Gibco, Rockville, MD, USA; and R&D Systems, Minneapolis, MN, USA. The RGCs were then incubated in HG medium in which 25 mM glucose replaced 5 mM glucose for 48 h as in vitro diabetic model. IL-17A (50 or 100 ng/ml) was applied to the RGC cultures for 24 h. The RGCs and supernatants were collected, respectively, for subsequent analysis.

### Transwell coculture of RMCs and RGCs

The purified RMCs that had been treated with IL-17RA-shRNA or Scr-shRNA were seeded on a membrane with 0.4 µM pores in a transwell insert (Millipore, Billerica, MA, USA) with Neurobasal medium. The transwell insert with RMCs was placed into a well of culture plate containing RGCs that had been cultured for 5 days. The ratio of the two cell types was 1:1. These cells were cocultured for 48 h in HG medium, and then IL-17A of 50 ng/ml was added to the transwell coculture plate, which was incubated for 24 h. The RMC inserts were removed, and the supernatants and RGCs underneath the transwell inserts were collected for further analysis.

### Western blot analysis

Retinas or cells were isolated and homogenized in radio immunoprecipitation assay buffer. Proteins (30 µg/lane) were separated by 12% sodium dodecyl sulfate–polyacrylamide gel electrophoresis and electroblotted to polyvinylidene fluoride membranes (Millipore, Temecula, CA, USA). The membranes were incubated with primary antibodies against occludin (#ab216327; 1:2000), zonula occludens-1 (ZO-1; #ab216880; 1:50), GFAP (#ab7260; 1:10,000), VEGF (#ab214424; 1:1000), GS (#ab176562; 1:1000), EAAT1 (#ab181036; 1:1000), caspase-3 (#ab184787; 1:1000), or caspase-9 (#ab202068; 1:1000) (all from Abcam, Cambridge, UK) overnight at 4 °C. After being washed, the membranes were incubated with IRDye 800-conjugated goat anti-rabbit IgG (LI-COR Inc., Lincoln, NE, USA, #926-32211; 1:5000) for 1 h at room temperature. The protein band intensities were quantified by densitometric analysis using the Image J software and expressed as relative values normalized to β-actin.

### Enzyme-linked immunosorbent assay (ELISA)

Retinas were homogenized ultrasonically in radio immunoprecipitation assay buffer supplemented with protease inhibitors in an ice water bath. The lysates were centrifuged at 10,500 × *g* for 15 min at 4 °C. The supernatants were tested for GFAP and VEGF levels with ELISA kits (Millipore, Billerica, MA, USA and R&D systems, Minneapolis, MN, USA, respectively) and the levels of GFAP and VEGF were normalized to the contents in retinal total proteins that were determined by bicinchoninic acid protein assay. For cultured RMCs, proteins were extracted from the cells and subjected to ELISA to determine GFAP content, and supernatants of the cells were directly tested by ELISA to determine VEGF concentration.

### High-performance liquid chromatography

Retinas were homogenized in 0.1 M perchloric acid at 4 °C. The extracts were centrifuged at 13,600 × *g* for 20 min at 4 °C and the supernatants were measured by a Nova-Pak C_18_ column (3.9 × 150 mm, 4 μm beads) for glutamate levels. Quantification was performed by running standard amino acid solutions under the same conditions.

### Immunofluorescence staining

Frozen retinal sections were prepared and incubated with mouse antibody against NeuN (Abcam, Cambridge, UK, #ab104224; 1:200) at 4 °C overnight. The sections were then stained with the secondary antibody anti-mouse Alexa Fluor 594 (Abcam, Cambridge, UK, #ab150116; 1:200) for 2 h at room temperature, followed by 4′,6-diamidino-2-phenylindole (DAPI) staining of 5 min to label cell nuclei.

RMCs and RGCs seeded onto coverslips were fixed with 4% paraformaldehyde for 20 min. The following primary antibodies were used: mouse anti-GS (#ab64613; 1:200), rabbit anti-IL-17RA (#ab180904; 1:200), mouse anti-NeuN (#ab104224; 1:200) (all from Abcam, Cambridge, UK), and chicken anti-NeuN (Novus Biologicals, Littleton, CO, USA, #NBP2-10491; 1:200). Alexa Fluor 488 (#ab150113), 594 (#ab150080), or 405 (#ab175674) conjugated secondary antibodies (all from Abcam, Cambridge, UK; 1:200) were followed to incubate these cells. In RMCs, GS^+^ cells and GS^+^IL-17RA^+^ cells in five visual fields of a coverslip were calculated into an average number, respectively. The average number of GS^+^IL-17RA^+^ cells in a coverslip was reported as a percentage of the average number of GS^+^ cells in statistical analysis.

### TdT-mediated dUTP nick end labeling (TUNEL) assay

The retinal sections stained with NeuN and DAPI were tested by TUNEL according to the manufacturer’s protocol (Roche Diagnostics, Indianapolis, IN, USA). The number of TUNEL^+^DAPI^+^ cells in three visual fields near retinal central area in one retinal section was calculated into a sum, and an average of TUNEL^+^DAPI^+^ cells in three sections of one retina was reported as a value in statistical analysis. The RGCs labeled with NeuN were also analyzed by TUNEL. Percentage of TUNEL^+^ cells in the sum of TUNEL^+^ and NeuN^+^ cells was calculated in a RGC coverslip, and an average percentage of five visual fields of a coverslip was reported as a statistical value.

### Lactate dehydrogenase (LDH) cytotoxicity assay

Supernatants of cultured RGCs were collected and measured by the LDH Assay Kit (BioVision, Mountain View, CA, USA) according to the manufacturer’s protocol. The absorbance plate reader (BioTek, Winooski, VT, USA) was used to read the amount of LDH release from the RGCs.

### SYTO-13/propidium iodide (PI) staining

RGCs were incubated with 0.1 µM SYTO-13 (Molecular Probes, Eugene, OR, USA) and 10 μg/ml PI (Sigma-Aldrich, St. Louis, MO, USA) in PBS at 37 °C for 5 min. Percentage of PI^+^ cells in the sum of SYTO-13^+^ and PI^+^ cells was calculated, and an average percentage of five visual fields of one slide was reported as a statistical value.

### Statistical analysis

All data are expressed as mean ± standard deviation. Sample sizes were estimated on the basis of previous similar experiments in the published papers and statistical concept on small sample size. Statistical analyses were performed with Statistics Package for Social Science (SPSS, 12.0). Comparisons between two groups were assessed using Student’s *t* tests. Multiple comparisons among the groups were evaluated by one-way analysis of variance, followed by post hoc analysis. The tests were two-sided. The variance was similar between the groups that were statistically compared. The results were considered statistically significant at *P* < 0.05.

## Results

### Retinal microvascular lesions are alleviated in IL-17A-deficient diabetic mice

**Fig. 1:**
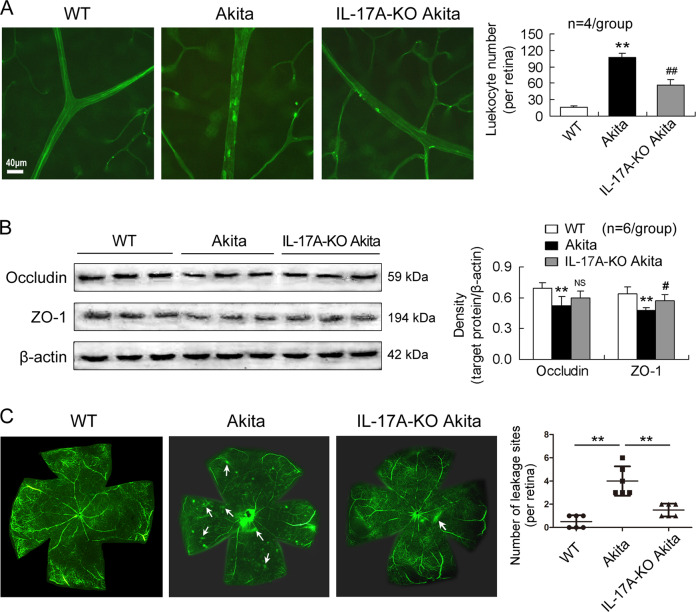
Retinal microvascular lesions are alleviated in IL-17A-deficient diabetic mice. Male Ins2^+/−^ (Akita) mice 12 weeks after the onset of diabetes and male Ins2^+/−^/IL-17A^−/−^ (IL-17A-KO Akita) mice at the age of 16 weeks were employed in the experiments. Age-matched male WT mice were used as a control of Akita mice and age-matched male Akita mice served as a control of IL-17A-KO Akita. **A** IL-17A gene loss reduces adherent leukocytes in the retinal vessels of DR. The adherent leukocytes were labeled by FITC-conjugated concanavalin A. Total number of adherent leukocytes in vessels per retina was statistically analyzed. The data of each group were obtained from four mice. **B** IL-17A gene deletion upregulates the retinal expression of ZO-1 protein in Akita mice. The data in the statistical histogram were from six mice for each group. **C** Retinal angiography showing that vascular leakage is mitigated in IL-17A-KO Akita mice. FITC-labeled dextran was perfused and the retinas were dissected and flat mounted. The leakage sites were counted in the retinal angiography by fluorescence microscopy. The arrows indicate typical FITC-dextran extravasations. **P < 0.01, versus WT mice; ^#^*P* < 0.05, ^##^*P* < 0.01, versus Akita mice; NS, no significance versus Akita mice.

As expected, retinas of Akita mice 12 weeks after the onset of diabetes showed increased number of leukocytes adhered to blood vessels (Fig. 1A), downregulated expression of the tight junction proteins occludin and ZO-1 (Fig. 1B), and enhanced dextran leakage from vessels (Fig. 1C), compared with respect to those of WT mice. Importantly, compared with Akita mice, IL-17A-KO Akita mice manifested a decrease in the number of adherent leukocytes in retinal vessels (Fig. 1A), an upregulation of retinal ZO-1 expression (Fig. 1B), and an attenuation of retinal vascular leakage (Fig. 1C).

### IL-17A deficiency mitigates RMC activation and dysfunction in diabetic mice

**Fig. 2:**
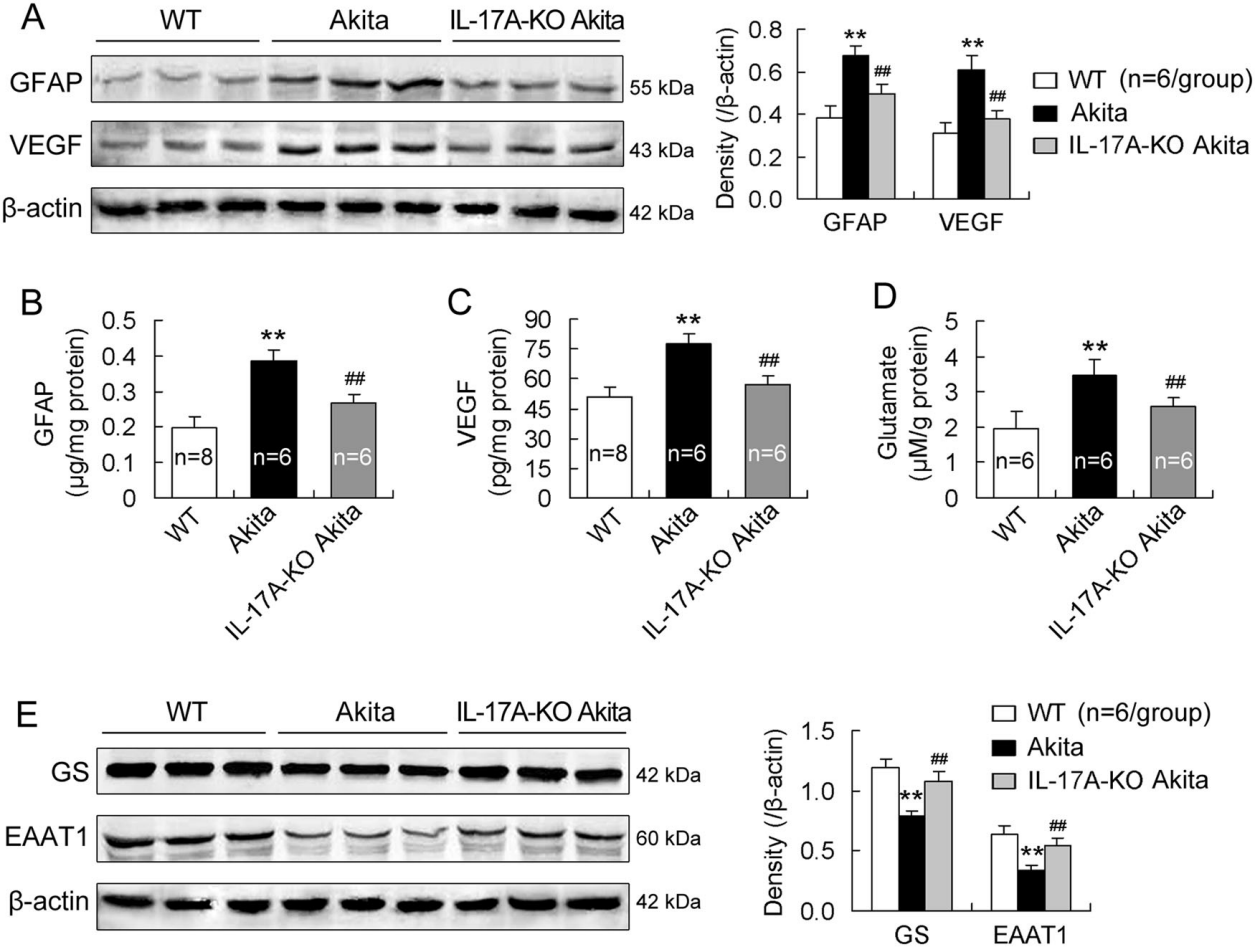
IL-17A deficiency mitigates RMC activation and dysfunction in diabetic mice. The design of the mice was similar to that in Fig. 1. **A** Expression of GFAP and VEGF in the retina. The data in the statistical histogram were from six mice for each group. **B**, **C** GFAP and VEGF contents in the retina. The numbers in the statistical histogram indicate that eight or six mice were used in the groups. **D** Glutamate content in the retina detected by HPLC. The numbers in the statistical histogram represent that six mice were used in each group. **E** Retinal expression of GS and EAAT1. The data from six mice for each group were statistically analyzed. **P < 0.01, versus WT mice; ^##^*P* < 0.01, versus Akita mice.

As indicated in Fig. 2A, retinal GFAP and VEGF expression in diabetic mice was upregulated relative to that in WT mice. Consistently, retinal GFAP and VEGF contents were higher in diabetic mice than in WT mice (Fig. 2B, C). Notably, both the expression and the contents of GFAP and VEGF in the retina were reduced in IL-17A-deficient diabetic mice in comparison to those in diabetic mice (Fig. 2A–C).

Unlike GFAP and VEGF, GS and EAAT1 were downregulated in the retinas of diabetic mice compared with those of WT mice (Fig. 2E). Corresponding to the downregulated GS and EAAT1 expression, glutamate content was increased in diabetic retinas relative to that in WT retinas (Fig. 2D). IL-17A gene loss compromised both the downregulated GS and EAAT1 expression and the increased glutamate level in the retinas of diabetic mice (Fig. 2D, E).

### IL-17A-deficient diabetic mice show a decreased RGC apoptosis

**Fig. 3:**
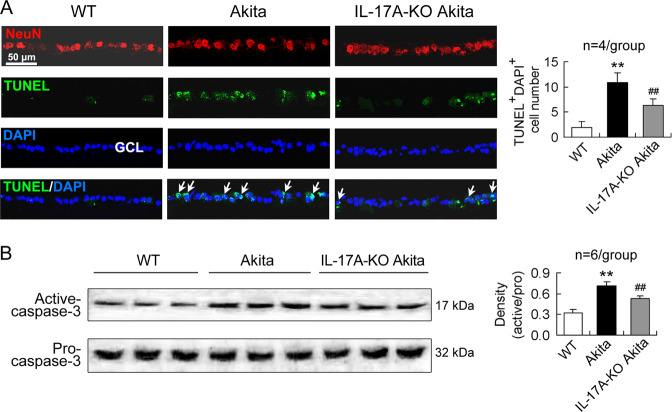
IL-17A-deficient diabetic mice show a decreased RGC apoptosis. The design of the mice was similar to that in Fig. 1. **A** Neuronal apoptosis in the GCL of the retina determined by TUNEL assay. The cells labeled by NeuN in the GCL indicate neurons. The TUNEL/DAPI double positive cells in the GCL represent apoptotic RGCs. The arrows indicate typical apoptotic RGCs. In the statistical histogram, TUNEL^+^DAPI^+^ cell number was obtained by averaging the number of three sections of one retina. The experiment was repeated four times. **B** Retinal caspase-3 activity analyzed by western blot. The statistical data were representative of six mice for each group. **P < 0.01, versus WT mice; ^##^*P* < 0.01, versus Akita mice.

Immunofluorescence staining of the retina with NeuN confirmed the neurons in the GCL (Fig. 3A). Simultaneously, staining the retina with DAPI indicated total cells in the GCL (Fig. 3A). Further, TUNEL assay assessed apoptotic cells in the GCL (Fig. 3A). The number of TUNEL/DAPI double positive cells in the GCL was increased in diabetic mice relative to that in WT mice (Fig. 3A). More importantly, IL-17A-KO diabetic mice showed a decrease in the number of TUNEL/DAPI double positive cells in the GCL compared with diabetic mice (Fig. 3A). Furthermore, caspase-3 activity was enhanced in the retinas of diabetic mice (Fig. 3B). The diabetes-induced caspase-3 activity in the retinas was attenuated by IL-17A gene ablation (Fig. 3B).

### RMCs express IL-17RA and silencing IL-17RA gene in RMCs abolishes IL-17A deleterious effects

**Fig. 4:**
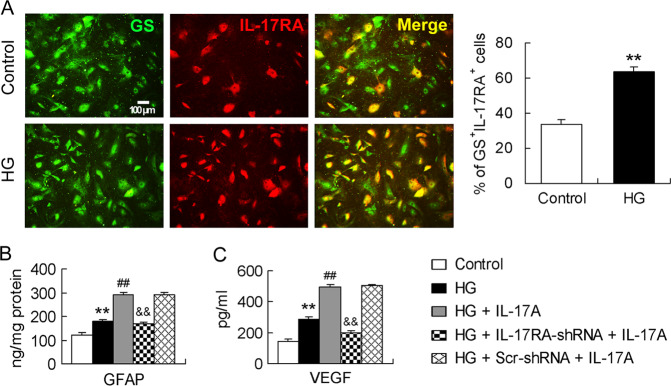
RMCs express IL-17RA and silencing IL-17RA gene in RMCs abolishes IL-17A deleterious effects. **A** HG increases IL-17RA expression in RMCs. Primarily cultured RMCs were treated with HG (25 mM) for 48 h and marked with GS. The percentage of GS^+^IL-17RA^+^ cells in total GS^+^ cells was reported in the statistical histogram and the data were from six independent experiments. **B** GFAP content in cultured RMC lysates measured by ELISA. The data were representative of five separate experiments. **C** VEGF concentration in cultured RMC supernatants determined by ELISA. The experiment was repeated eight times. **P < 0.01, versus control; ^##^*P* < 0.01, versus HG; ^&&^*P* < 0.01, versus HG + Scr-shRNA + IL-17A.

GS, a marker of RMCs, was co-localized with IL-17RA in primarily cultured RMCs (Fig. 4A). Percentage of GS^+^IL-17RA^+^ cells in total GS^+^ cells was higher in HG condition than in control (Fig. 4A). HG treatment increased GFAP and VEGF production in RMCs and IL-17A exposure further increased HG-induced GFAP and VEGF production (Fig. 4B, C). However, RMCs in which IL-17RA gene was knocked down with IL-17RA-shRNA no longer manifested the increased GFAP and VEGF production in response to IL-17A compared with those cells with Scr-shRNA treatment (Fig. 4B, C).

### RGCs do not express IL-17RA and IL-17A does not directly exacerbate HG-induced neuronal death

**Fig. 5:**
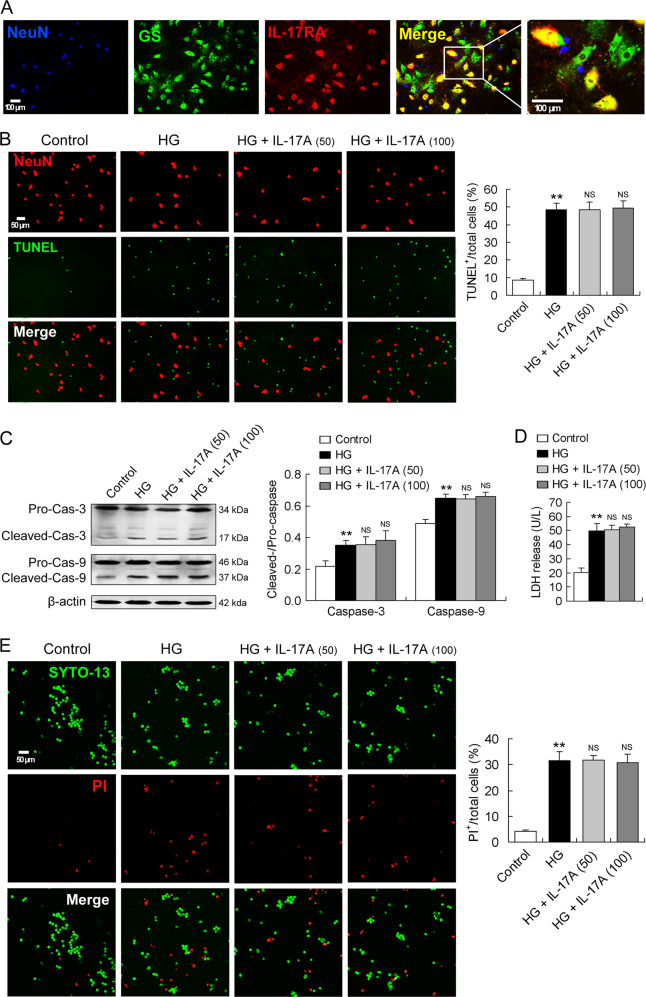
RGCs do not express IL-17RA and IL-17A does not directly exacerbate HG-induced neuronal death. **A** Immunofluorescence images showing IL-17RA expression. Cocultures of RMCs and RGCs were stained with NeuN, GS, and IL-17RA to compare and identify the expression of IL-17RA in the two types of cells. As indicated in Fig. 4, GS and IL-17RA were co-localized. However, no co-localization of NeuN and IL-17RA was observed in the cocultures. **B** TUNEL assay showing RGC apoptosis. IL-17A was applied to primarily cultured RGCs at a concentration of 50 ng/ml (50) or 100 ng/ml (100) for 24 h in HG condition. **C** Caspase-3 and caspase-9 active levels determined by western blot analysis. **D** LDH release levels in RGCs. **E** SYTO-13/PI double staining showing RGC necrosis. Percentage of PI^+^ dead cells in the sum of SYTO-13^+^ viable cells and PI^+^ dead cells was registered for the statistics. All the data in the statistical histograms were obtained from five independent experiments. **P < 0.01, versus control; NS, no significance versus HG.

Cocultures of RGCs and RMCs were stained with NeuN, GS, and IL-17RA to compare and identify IL-17RA expression in the two types of cells. As shown above, GS was co-localized with IL-17RA (Fig. 5A). In contrast, no co-localization of NeuN with IL-17RA was observed in the cocultures (Fig. 5A).

HG raised percentage of TUNEL^+^ cells in the sum of NeuN^+^ and TUNEL^+^ cells in primarily cultured RGCs (Fig. 5B). Interestingly, IL-17A (50 or 100 ng/ml) did not further alter HG-induced increase in TUNEL^+^ cell percentage in RGCs (Fig. 5B). Similarly, HG enhanced both caspase-3 and caspase-9 activities in RGCs, and IL-17A did not further alter HG-enhanced caspase-3 and caspase-9 activities (Fig. 5C).

LDH release level, which reflects cell necrosis, was elevated by HG in RGCs (Fig. 5D). Similarly, HG-induced LDH release from RGCs was not further affected by IL-17A (Fig. 5D). Furthermore, RGC necrosis was also assessed by SYTO-13/PI double staining. Percentage of PI^+^ cells in total cells including SYTO-13^+^ and PI^+^ cells was higher in HG-treated RGCs than in control cells (Fig. 5E). However, IL-17A treatment (50 or 100 ng/ml) did not further alter HG-induced increase in PI^+^ cell percentage (Fig. 5E).

### IL-17A exacerbates HG-induced RGC death in the presence of intact RMCs but not in the presence of IL-17RA-silenced RMCs

**Fig. 6:**
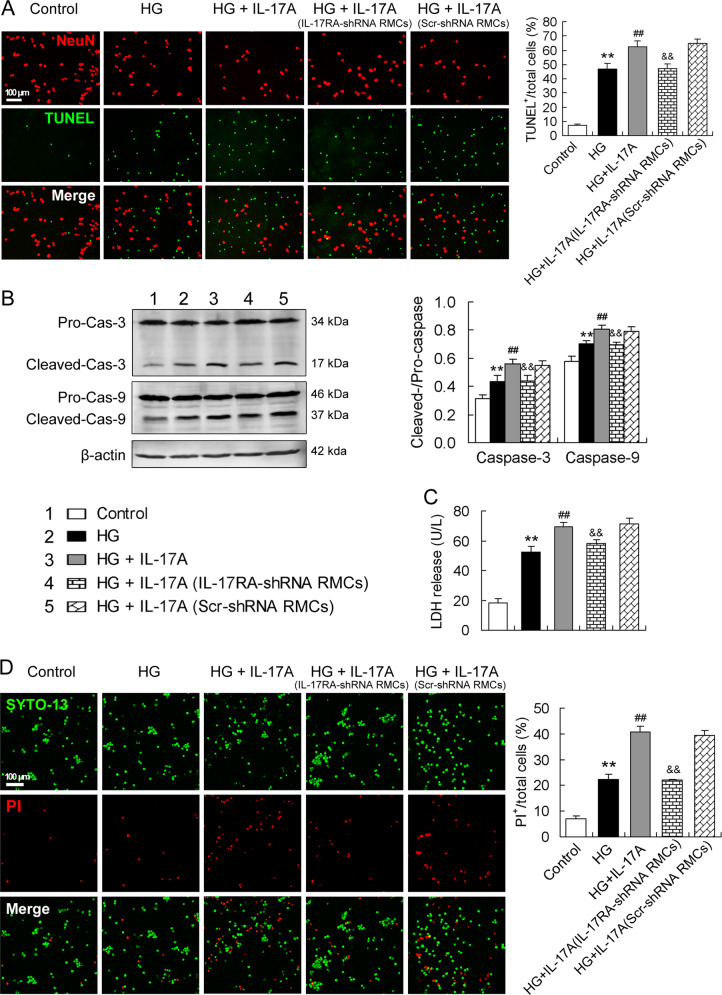
IL-17A exacerbates HG-induced RGC death in the presence of intact RMCs but not in the presence of IL-17RA-silenced RMCs. Transwell cocultures of RGCs and RMCs that had been treated with IL-17RA-shRNA or Scr-shRNA (as a control) were exposed to IL-17A (50 ng/ml) in HG condition for 24 h and the cocultured RGCs were analyzed. **A** NeuN staining and TUNEL assay of the cocultured RGCs. Apoptosis of the RGCs was assessed by the percentage of TUNEL-positive cells in total cells including NeuN-positive and TUNEL-positive cells. **B** Active levels of caspase-3 and caspase-9 in the cocultured RGCs. **C** LDH release levels showing RGC necrosis. **D** SYTO-13/PI double staining of the RGCs. Percentage of PI-positive cells in the sum of SYTO-13-positive and PI-positive cells was reported in the statistical histogram. All the data in the statistical histograms were from five separate experiments. **P < 0.01, versus control; ^##^*P* < 0.01, versus HG; ^&&^*P* < 0.01, versus HG + IL-17A (Scr-shRNA RMCs).

The transwell cocultures of RGCs and RMCs that had been treated with IL-17RA-shRNA or Scr-shRNA (as a control) were exposed to IL-17A (50 ng/ml) in HG medium and then the cocultured RGCs were analyzed. Compared with control, HG treatment increased TUNEL-positive cell percentage in the cocultured RGCs (Fig. 6A). Importantly, IL-17A exposure enlarged HG-induced increase in TUNEL-positive cell percentage in the cocultured RGCs (Fig. 6A). More importantly, IL-17A no longer enlarged HG-induced increase in TUNEL-positive cell percentage in RGCs when the cells were cocultured with RMCs whose IL-17RA gene had been silenced with IL-17RA-shRNA (Fig. 6A). Similarly, HG-enhanced caspase-3 and caspase-9 activities were further increased by IL-17A in the cocultured RGCs (Fig. 6B). However, HG-enhanced caspase-3 and caspase-9 activities were not further altered by IL-17A when the RGCs were cocultured with RMCs in which IL-17RA gene had been knocked down (Fig. 6B).

In the same way, IL-17A further increased HG-induced LDH release from RGCs when the cells were cocultured with Scr-shRNA-treated RMCs but not with IL-17RA-shRNA-treated RMCs (Fig. 6C). Furthermore, HG-induced PI-positive RGC increase was further elevated by IL-17A when the cells were cocultured with Scr-shRNA-treated RMCs but not with IL-17RA-silenced RMCs (Fig. 6D).

## Discussion

IL-17A-deficient Akita mice manifested the reduced vascular leukostasis, ZO-1 downregulation, and vascular leakage in the retina in the current study. This demonstrates that IL-17A is actively implicated in retinal microvascular lesions, a major characteristic for DR. Recently, other studies have shown that retinal endothelial cells express IL-17RC [12] and that IL-17A induces retinal endothelial cell death and therefore enhances retinal capillary degeneration [35]. These other authors’ findings suggest a direct damage of IL-17A to retinal vessels. The increased vascular permeability by IL-17A may promote infiltration of proinflammatory lymphocytes and cytokines including Th17 cells and IL-17A into retinal tissue from vasculature and lead to DR progression. Therefore, IL-17A gene deletion in this study mitigates retinal microvascular lesions and DR pathophysiology. We previously showed that blood glucose concentration was decreased in IL-17A-KO Akita mice from age 10 weeks to 16 weeks (the last monitored age) compared with that of Akita mice [32]. Collectively, these results suggest that the mitigated retinal microvascular lesions by IL-17A deficiency are related to the decreased blood glucose concentration. However, the body weight of IL-17A-KO Akita mice was not significantly different from that of Akita mice (data were not listed), suggesting that body weight is not a predominantly affected factor by IL-17A.

Diabetes is associated with RMC activation that is characterized by GFAP increase in the cells [19, 36]. RMCs regulate retinal inflammation, neovascularization, and vascular leakage and lesion, the key DR-associated pathological features by producing a major angiogenic factor, VEGF [20]. RMCs take up extracellular glutamate through EAAT1 and convert it into glutamine via GS, and by this mechanism glutamate removal and prevention of neurotoxicity in the retina is achieved [19, 37]. We showed that IL-17A deficiency ameliorated retinal abnormalities related to RMC activation, VEGF production, and glutamate uptake and metabolism. The results suggest that IL-17A is actively involved in RMC activation and dysfunction during DR. Further, in vitro study supports the in vivo findings. Primarily cultured RMCs expressed IL-17RA and this expression was upregulated in HG condition. IL-17A enhanced HG-induced GFAP and VEGF production in RMCs. However, silencing IL-17RA gene with IL-17RA-shRNA in RMCs abolished the IL-17A effect. IL-17A exerts its effect by binding to a heterodimeric receptor complex of IL-17RA and IL-17RC [13]. Lack of either IL-17RA or IL-17RC in the heterodimer completely abrogates IL-17 function [38]. Thus, we propose that IL-17A promotes RMC activation and dysfunction by activating IL-17RA expressed on the cells. The IL-17A-related intracellular signaling pathway (Act1-TRAF6-IKK-NFκB) has been shown to mediate IL-17A impairment to RMCs in our previous work [10]. Here we identify that IL-17RA is expressed by RMCs and it mediates IL-17A injury to the cells during DR process.

In the course of diabetes, there is a chronic loss of retinal neurons due to increased frequency of apoptosis and the neuronal apoptosis begins very early [2, 5, 39–41]. In this study, IL-17A gene loss reduced RGC apoptosis in Akita diabetic mice, suggesting that IL-17A is involved in retinal neuronal apoptosis of DR. It has been reported that IL-17A^−/−^ reduces diabetes-mediated retinal inflammation, oxidative stress, and vascular leakage in streptozotocin-induced diabetic mice [12]. Our present results provide further evidence showing that retinal neuronal apoptosis is also attenuated by IL-17A gene ablation. Interestingly, in vitro study indicated that RGCs did not express IL-17RA and IL-17A did not further alter HG-induced RGC death. The data suggest that IL-17A does not directly affect RGC survival. Neuronal expression of IL-17 receptors is controversial [14–17]. This implies a possibility that, in different animal species and in different stages of neuronal differentiation and development, IL-17 receptor expression may undergo a change [42]. However, in vivo study reveals that IL-17A deficiency can affect RGC apoptosis. It suggests a possibility that other retinal cells mediate the effect of IL-17A on RGC apoptosis. RMCs are the most likely retinal cells that transmit IL-17A proinflammatory information to RGCs, in view of the evidence that RMCs are directly affected by IL-17A and have a close connection with RGCs.

Indeed, IL-17A treatment of the cocultures of RGCs and RMCs aggravated HG-induced RGC death. However, IL-17A treatment of the cocultures of RGCs and RMCs in which IL-17RA gene had been silenced no longer aggravated HG-induced RGC death. These findings imply that RMCs are required for IL-17A injury to RGCs. It suggests that IL-17A directly damages RMCs by activating IL-17RA expressed on the cells and then kills RGCs via RMC mediation. RGCs are enveloped by RMC processes [43] and also RMCs enclose many neuronal somata and processes [44]. The extensive contact of RMCs with retinal neurons allows RMCs to actively modulate and support retinal neuronal function and survival in physiological and pathological responses. Accordingly, it is reasonable that RMCs convey IL-17A inflammatory information to RGCs to affect their survival by some mechanisms. The mechanisms may involve a cell-to-cell contact communication or/and a mediator-mediated crosstalk between RMCs and RGCs. In addition, other types of cells, such as microglia, probably also participate in mediating IL-17A damage to RGCs. It has been reported that IL-17A exacerbates dopaminergic neuronal loss only in the presence of microglia and IL-17A-treated microglial medium facilitates dopaminergic neuronal death, suggesting that IL-17A accelerates neurodegeneration in Parkinson’s disease depending on microglial activation [42]. Further evidence revealing the mechanisms of IL-17A affecting RGC survival in DR process remains to be provided in the future studies.

In summary, IL-17A-KO diabetic mice show alleviated retinal microvascular lesions, RMC activation and dysfunction, and RGC apoptosis. The results demonstrate that the proinflammatory cytokine IL-17A is actively implicated in DR pathophysiology. Although Th17 cells are a major source of IL-17A, other cell types, such as innate immune cell populations [45], microglia [46], astrocytes [47], and RMCs [10], can also produce IL-17A. Thus, the effects of global IL-17A gene deletion on DR pathophysiology may be an integrative outcome of the multiple cell changes. Further, in vitro study identifies that IL-17A directly impairs RMCs by activating IL-17RA expressed on the cells. In contrast, RGCs do not express IL-17RA and IL-17A does not directly affect RGC survival but rather depends on RMCs to kill the neurons. These findings establish that IL-17A promotes diabetes-induced RGC death via RMC mediation. Neurodegeneration cannot be reversed and therefore treatments preventing neuronal cell loss in the retina need to be developed to protect diabetic patients [2]. We propose that blocking IL-17RA on RMCs may develop a prospective therapeutic strategy to prevent neuronal death in DR progression.

## Data Availability

All data generated or analyzed during this study are included in this published article.
